# Hepatitis B infection among HIV infected individuals in Gabon: Occult hepatitis B enhances HBV DNA prevalence

**DOI:** 10.1371/journal.pone.0190592

**Published:** 2018-01-09

**Authors:** Berthold Bivigou-Mboumba, Marie Amougou-Atsama, Samira Zoa-Assoumou, Hervé M’boyis Kamdem, Guy Francis Nzengui-Nzengui, Angélique Ndojyi-Mbiguino, Richard Njouom, Sandrine François-Souquière

**Affiliations:** 1 Unité Mixte de Recherches VIH et Maladies Infectieuses Associées (UMR VIH-MIA), Centre International de Recherches Médicales (CIRMF), Libreville, Gabon; 2 Laboratoire de Bactériologie-Virologie, Laboratoire de Référence IST-SIDA, Université des Sciences de la Santé, Owendo, Gabon; 3 Service de Virologie, Centre Pasteur du Cameroun, Yaoundé, Cameroun; Centers for Disease Control and Prevention, UNITED STATES

## Abstract

In Gabon, a central African country, human immunodeficiency virus (HIV) and hepatitis B virus (HBV) are endemic. In a recent study, conducted in a semi-urban area (Franceville, Gabon), HBV infection was found to be more prevalent among HIV infected individuals. This study aims to investigate the prevalence and genetic diversity of hepatitis B virus infection among HIV infected individuals, predominantly under antiretroviral therapy, living in fully urbanized area: Libreville, capital of Gabon. Serological and molecular tests were performed to detect HBV infection among patients living with HIV/AIDS (PLHA). We used Monolisa HBsAg ULTRA, Anti-HBc Plus and Anti-HBs Plus EIA kits for serological analyses. HBV DNA viral load (HBV DNA VL) was determined by real time PCR and molecular characterization of HBV strains was performed by sequencing and phylogenetic analysis of partial HBV surface and core genes. At all, 70.2% of patients were under antiretroviral therapy. The prevalence of HBsAg was 8.8% (43/487). Detectable HBV DNA was found in 69.7% (30/43) of HBsAg positive patients and in 17.5% (24/137) HBsAg negative patients. HBV DNA VL was significantly higher among patient with CD4 cell counts less than 200 cells/mm^3^ than those with CD4 cell counts greater than 500 cells/mm^3^ (*p* = 0.008). We confirmed the presence of HBV sub-genotypes QS-A3 (40%), and A4 (20%) and HBV-E genotype (40%). The percentage of resistance to Lamivudine was high (40%) and varied according to the M204V/I motif. Occult hepatitis B infection (OBI) was found in patients with isolated HBcAb and among patients who had completed their HBsAg seroconversion. We detected HBV DNA for one patient without any HBV serological marker. This study provides a new landmark for the comprehension of HBV infection in PLHA in urban areas. OBI enhances HBV DNA prevalence and should be investigated in all HBsAg negative individuals.

## Introduction

The emergence of chronic infection due to Hepatitis B Virus (HBV), in the context of immunosuppression in patients infected with Human immunodeficiency virus (HIV), remains a public health problem. According to the World Health Organization (WHO-2015), nearly 36.7 million people in the world, are living with HIV/AIDS (PLHA) and 2/3 of those are living in Sub-Saharan Africa [1]. In addition, this area is hyper endemic for HBV with an exposure rate up to 90% [2]. Around 257 million people living with hepatitis B and in 2015 around 1.34 million people die of viral hepatitis, near to HIV death rate (WHO-2017). Due to its highly contagious capacity and some transmission routes in common with HIV, HIV-HBV co-infection is frequent in resource-limited settings (RLS), where 10% of HIV infected are co-infected with HBV [3]. Chronic HBV infection is responsible for about 60% of the total liver cancer in RLS [4]. Studies highlight the negative impact of co-infection [5,6]. HIV favors the progression of HBV infection to cirrhosis and hepatocellular carcinoma [7]. Studies show that HIV promotes an expansion of HBV DNA VL and increases the risk of liver-related disease particularly among patients with low CD4 cell counts (less than 200 cells/mL) [8,9]. Thus, HIV/HBV co-infection negatively impacts immunological recovery as compared to HBV mono-infection.

The new classification of HBV genotypes reveals 10 genotypes (from A to J). A redefinition of sub-genotypes was recently proposed with the creation of Quasi-sub-genotypes (QS) QS-A3, QS- B3, QS-C2, regrouping clusters in each genotype, and a recombino-subgenotype within the genotype D [10,11]. The relation between genotypes and clinical outcome is not clearly demonstrated, depending on the geographical area and pathology studied [12]. Therefore, HBV genotyping and mutational analyses are significant issues for enhanced monitoring of infected patients. For PLHA, the HBV diagnosis is essential, particularly before initiation of an antiretroviral regimen [13].

In Gabon, a Central African country, HBsAg prevalence in the general population varied between 8 to 10% [14]. Previous studies conducted among urban, rural and pregnant women populations showed HBsAg prevalences of 12.9%, 7.6% and 9.2% respectively [15,16]. All of them revealed the circulation of HBV-A and HBV-E genotypes and of the recombinant HBV strains (A/E, E/A, and D/E). In a more recent study we showed, in a semi-urban area (Franceville, Gabon), that HBsAg prevalence and HBV DNA were also high (9.2% and 17.4% respectively) in HIV infected patients [17]. Genotypes described were A and E, with a prevalence of QS-A3 HBV strains. A high rate (47.4%) of the mutation conferring resistance to Lamivudine (major mutation M204V/I) was registered [17].

This study was, thus, conducted to investigate the prevalence and genetic diversity of HBV infection among HIV infected individuals in Libreville, the fully urban capital city of Gabon. The results reported here allow us to evaluate the percentage of HIV-HBV co-infection and the quality of the therapeutic management of hepatitis B in this context.

## Materials and methods

### Study design, participants and sample collection

This cross-sectional study was conducted among HIV infected patients living in Libreville, Gabon between October 2015 and April 2016. We included all patients over the age of 15 years, who recorded an HIV viral load during the study period. They were consecutively recruited during their visit to the HIV care centers and were predominantly under antiretroviral therapy. Their informed consent was obtained regarding the main objective of this study based on the monitoring of HIV and hepatitis B infected patients. Five milliliters of whole blood were collected in EDTA tubes from which 2 aliquots of plasma were obtained and stored at -20°C for subsequent analyses. The study was approved by the Ethics Committee of Gabon (N°0021/2013/SG/CNE). Written informed consent was obtained from each patient.

### Laboratory analyses

#### • HBsAg, anti-HBc and anti-HBs antibody screenings

HBsAg, anti-HBc and anti-HBs screenings were performed using the Monolisa HBsAg ULTRA, Monolisa Anti-HBcPlus, and Monolisa Anti-HBs Plus kits (Bio-Rad, Marnes-La-Coquette-France), respectively, as described previously [18]. All the tests were conducted according to the manufacturer’s instructions.

#### • CD4+ T cell counts

A total of 50 μL of whole blood was used to perform CD4+ T cell counts, using BD FACSCount^™^ CD4 Reagents as recommended by the manufacturer (BD Biosciences France Le Pont de Claix, France) [19].

#### • HBV DNA quantification

HBV quantification was performed in all HBsAg positives and some samples HBsAg negatives, because of the availability of HBV DNA quantitative regents. For this, we used the AcroMetrix HBV Panel (Menica, CA, USA), primers/probe set designed under ANRS 12187 project and Platinum UGD Master Mix (ThermoFisher, Waltham, Massachusetts, USA), as previously described [17]. The lower limit of detection with this technique was 50 IU/mL http://dx.doi.org/10.17504/protocols.io.kw4cxgw.

#### • HBV genotyping

HBV genotyping was performed by sequencing and phylogenetic analyses of the surface (S) and core (C) fragments. Briefly, HBV DNA was first extracted from 400 μL of plasma and eluted in 100 μL of pure water, using the QIAamp Viral DNA Mini Kit (QIAGEN, Courtaboeuf, France) followed by semi-nested PCR amplification of the S (930 bp) and C (1010 bp) gene fragments using MP *Taq* Core Kits 25 (MP Biomedical Diagnostic, Europe) http://dx.doi.org/10.17504/protocols.io.kyhcxt6. The S fragment amplification was performed as described elsewhere [20–22]. The first round was performed using primers sets 58P (5’-CCT GCT GGT GGC TCC AGT TC-3’) and 979 (5’-ATT GGA AAG TAT GTC AAA GAA TTG TGG GTC TTT TG-3’). The 50 μL final reaction mixture contained 31.4 μL of RNase DNase Free water, 5 μL of buffer 10X with MgCl_2_ (25 mM), 0.4 μL of dNTPs (25 mM), 1.5 μL of each primer (10 μM), 0.2 μL of *Taq* polymerase (5 U/μL) and 10 μL of extracted DNA. The second round PCR used the 58P/Mc2r (5’-TGGAAGTTGGGGATCATTGCC-3’) primer on a 50 μL final reaction mixture containing 36.4 μL of RNase DNase Free water, 5 μL of buffer 10X with MgCl_2_ (25 mM), 0.4 μL of dNTPs (25 mM), 1.5 μL of each primer (10 μM), 0.2 μL of *Taq* polymerase (5 U/μL), and 5 μL of first round PCR product. The PCR program was the same for the first and second round PCRs, including denaturation at 94°C for 5 min followed by 40 cycles of denaturation at 94°C for 1 min, annealing at 55°C for 30 s and elongation at 72°C for 1 min, followed by final elongation at 72°C for 5 min. For the C fragment amplification, the couple of primers BCP1F (5’-GCA TGG AGA CCA CCG TGA AC-3’) / 2853N (5’-TCA CCA TAT TCT TGG GAA CA-3’) was used for the first round and the couplet BCP2F (5’-CAT AAG AGG ACT CTT GGA CT-3’) / 2853N for the second round. The amplification conditions were the same as for the S fragment.

PCR products were purified using QIAquick PCR Purification Kit (Qiagen, Courtaboeuf, France) and submitted for sequencing at Macrogen Inc (Meibergdreef, Netherlands). Briefly, we Added 5 volumes of Buffer to 1 volume of the PCR sample and mix. QIAquick spin column was placed in a provided 2 ml collection tube and centrifuge for 60 s. To wash two times, we added 0.75 ml Buffer PE to the QIAquick column and centrifuge for 60 s. For elution of DNA, we added 50 μl of pure water (pH 7.0–8.5) to the center of the QIAquick membrane and centrifuge the column for 1 min (QIAquick PCR Purification Kit Protocol; available online: http://2012.igem.org/wiki/images/a/a3/QIAquick_PCR-purification.pdf).

### Sequence analyses

#### • Phylogenetic analyses

Phylogenetic analyses were performed to determine genotype and sub-genotype. Fasta file was generated using our sequences. The 87 reference sequences for HBV genotypes and sub-genotypes A to G were retrieved from GenBank. Pairwise alignments were performed using MEGA 7.0.14 software [23]. The phylogenetic tree was constructed by Bayesian inference using the Bayesian Markov chain Monte Carlo (MCMC) statistical framework implemented in Mr Bayes version 3.1.2 software as previously described [24].

#### • Mutational analysis

As previously described [17], we used the Mutation Reporter Tool (MRT) software (http://hvdr.bioinf.wits.ac.za/mrt/) [25] to look for HBV resistance-associated mutations (RAMs) in the Polymerase catalytic domain represented by major RAMs (A181T/V/S, A194T, M204V/I/S and N236T) and compensatory RAMs (I169T, V173L, L180M, S202G/I and M250V [26]. In addition, we looked for Vaccine Escape Mutants (VEMs) and polymorphic mutations outside (Y100C, Q101H, S117N, T118R and P120S) and within the HBsAg immuno-dominant ‘a’ determinant (I/T126A/N, A128V, Q129H/R, G130N, M133L/T, K141E, S143L, D144A/H/E and G145R) [27].

### Statistical analysis

Statistical analysis was performed using Statistica software (StatSoft France, 2005. STATISTICA, version 7.1. www.statsoft.fr). Prevalences were expressed with 95% confidence intervals (95% CI). Continuous data were expressed as median values with 1st and 3rd interquartile ranges (IQR). Comparisons between groups were conducted using the Mann-Whitney test, while categorical data were expressed as percentages and compared across groups using the χ2 test or Fisher exact test. The *p*-values were 2-sided and considered statistically significant when *p* <0.05.

## Results

### Baseline characteristics

A total of 487 PLHA were included in this cross-sectional study. Among them, 326 (66.9%) were women. The median age was 44 years old (IQR, 37–42 years old). The 30 to 45 years old age group was the largest (42.5%) (Table 1).

**Table 1:** Baseline characteristics and HBsAg prevalence in the studied population.

Variable	All patients (n = 487) n (%)	HBsAg^Pos^ Prevalence n (%)
**Gender**		**p = 0.05**^a^
Male	161 (33.1)	20 (12.4)
Female	326 (66.9)	23 (7.0)
**Ages**		**p = 0.06**^b^
[15–30]	34 (7.0)	0 (0.0)
[30–45]	213 (42.5)	16 (7.5)
[45–60]	194 (39.2)	20 (6.8)
[60 and More]	46 (9.4)	7 (15.2)
**CD4 Count (cells/mm**^**3**^**)**		**p = 0.27**^b^
CD4<200	191 (39.2)	22 (11.5)
200≤CD4<350	97 (20.0)	7 (7.2)
350≤CD4<500	80 (16.4)	4 (5.0)
CD4≥500	82 (16.8)	5 (6.0)
Missing	37 (7.6)	5 (11.6)

HBsAg: hepatitis B virus surface antigen; Pos: positive;

^a^: Chi-square test;

^b^: the student T-test; n: number of patients.

The mean CD4 count was 273 ± 243 cells/mm^3^. More than half of patients (59.2%) had less than 350 CD4 cells/mm^3^, including 39.2% with less than 200 CD4 cells/mm^3^.

**Fig 1:**
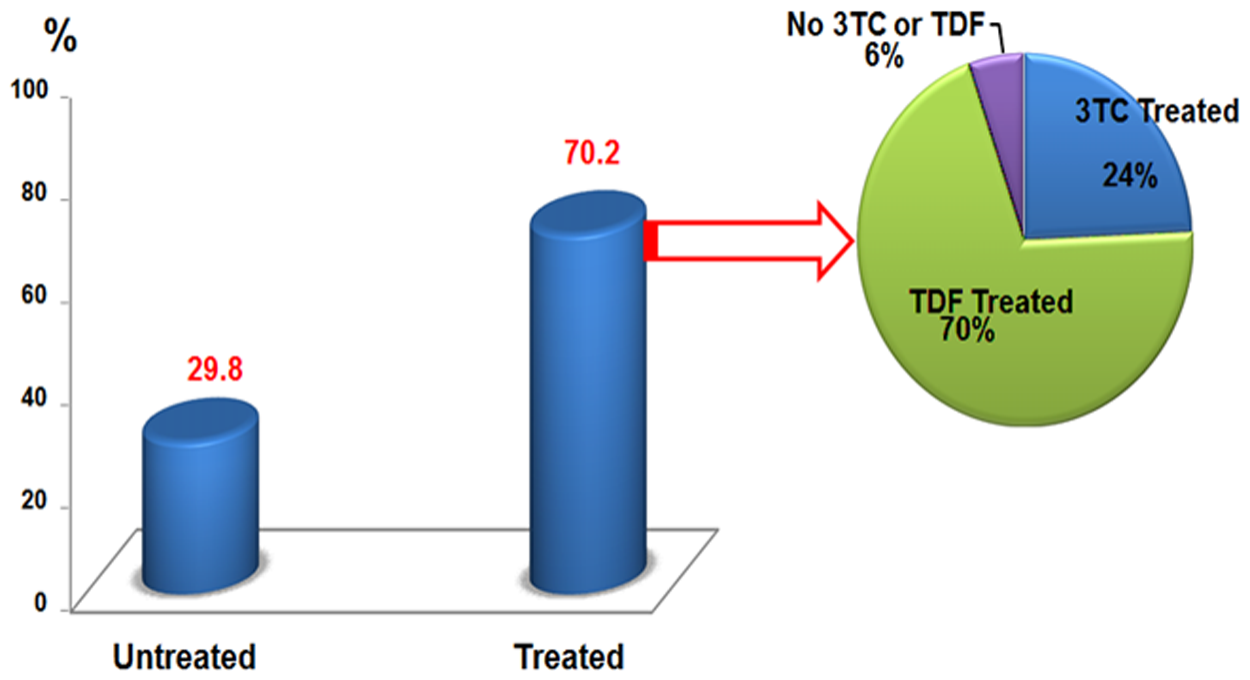
Percentage of patients untreated versus treated, and distribution of antiviral HBV therapies.

A majority of patients (70.2%) were on antiretroviral therapy (ART) for HIV infection. Tenofovir disoproxil fumarate (TDF) and Lamivudine (3TC) or Emtricitabine (FTC) was included in the regimen of 70% of them. The 3TC monotherapy was included in the regimen of 24% of patients. Finally, for the remaining 6% of patients neither the TDF nor 3TC were included in their regimen (Fig 1).

### HBsAg prevalence and HBV viral load

HBsAg was detected in 43/487 of PLHA (8.8%, 95%CI [6.8; 10.6]). This HBsAg prevalence was significantly higher in male (12.4%, 95%CI [7.5; 17.3]) than in female (7%, 95%CI [4.6; 9.4]) (p = 0.05). Although the highest HBsAg prevalence was found in the age group over 60 years (15.2%, 95%CI [4.8; 25.6]), the HBsAg prevalence was not significantly different according to age group (p = 0.06). The highest HBsAg prevalence was observed in patients with a CD4 count less than 200 cells/mm^3^ (11.5%, 95%CI [7.2; 15.8]). However, this prevalence was not statistically different with regard to the CD4 count level of patients (p = 0.27) (Table 1).

We performed HBV DNA VL on all 43 HBSAg positive (HBsAg^Pos^) and 137 HBsAg negative (HBsAg^Neg^) patients. Detectable HBV DNA was found in 69.7% (30/43) of HBsAg^Pos^ patients, and in 17.5% (26/137) of HBsAg^Neg^ patients. The highest prevalence of HBV DNA detection was found among HBV serological profile HBsAg^Pos^_HBcAb^Pos^, (69.7%, 95%CI [59; 80.4]) followed by the profile HBsAg^Neg^_HBcAb^Pos^_HBsAb^Neg^, (35%, 95%CI [20.2; 49.8]) and the profile HBsAg^Neg^_HBcAb^Pos^_HBsAb^Pos^ (16.4%, 95%IC [7.6; 25.3]). No HBV DNA was detectable in the HBsAg^Neg^_HBcAb^Neg^_HBsAb^Pos^ profile (Table 2).

**Table 2:** HBV DNA detection and HBV DNA VL according to HBV serological profiles.

HBV serological profiles	Number	HBV DNA^Pos^ % (n)	HBV DNA VL IU/mL	
			>2000 n (%)	<2000 n (%)
**HBsAg**^**Pos**^ **_ HBcAb**^**Pos**^	**43**	**69.8% (30)**	**12 (40)**	**18 (60)**
**HBsAg**^**Neg**^**_HBcAb**^**Pos**^ **_HBsAb**^**Pos**^	**70**	**16.4% (11)**	**1 (7.1)**	**10 (92.9)**
**HBsAg**^**Neg**^**_HBcAb**^**Pos**^**_HBsAb**^**Neg**^	**40**	**27.5% (11)**	**0 (0)**	**14 (100)**
**HBsAg**^**Neg**^; **HBcAb**^**Neg**^ **_HBsAb**^**Pos**^	**4**	**0.0% (0)**	**0 (0)**	**0 (0)**
**HBsAg**^**Neg**^ **_HBcAb**^**Neg**^ **_HbsAb**^**Neg**^	**23**	**15.4% (4)**	**0 (0)**	**1(100)**

HBV: hepatitis B virus; HBsAg: hepatitis B virus surface antigen; HBcAb: antibody against hepatitis B virus core antigen; HBsAb: antibody against hepatitis B virus surface antigen; Pos: positive; Neg: negative; HBV DNA: nucleic acid of hepatitis B virus; HBV DNA VL: viral load of nucleic acid of hepatitis B virus; n: number of profiles.

HBV DNA was detected in one patient without any HBV serological marker. This specimen was amplified in core gene by nested PCR (S1 Method). On the other hand, the highest HBV VL was also detected in the HBsAg^Pos^_HBcAb^Pos^ serological profile with about 40% of HBV VL > 2000 copies/mL.

**Fig 2:**
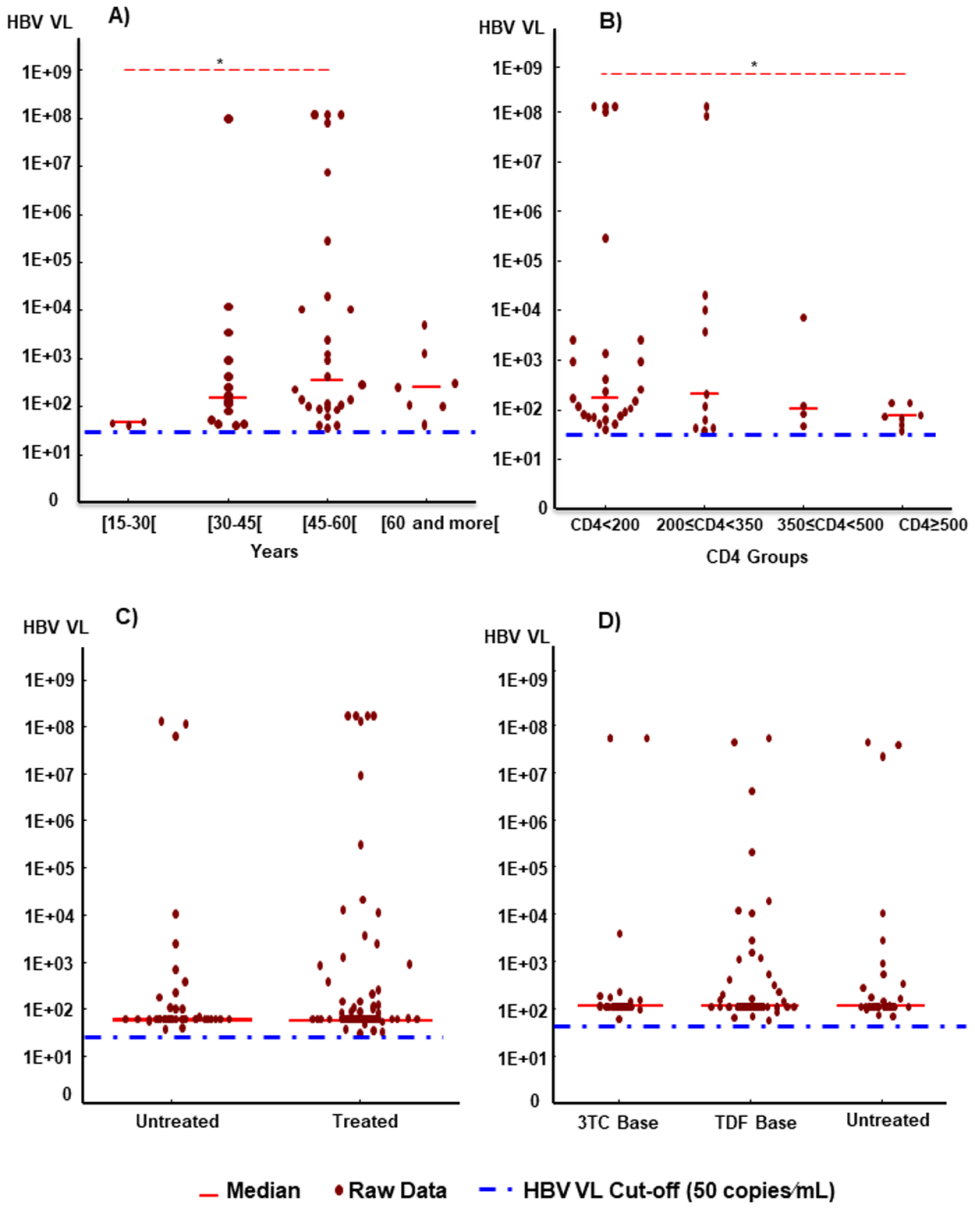
Comparative analysis of HBV DNA viral load according baseline characteristics of HIV positive patients. A) According to age group; B) According to CD4 count levels; C) and D) According to treatment status. Red line: Median; Red points: Raw data; Blue dash: HBV VL Cut-off (50 IU/mL).

HBV DNA VL spread through all four age groups, but patients from 15 to 30 years old had the lowest HBV DNA VL compared to the other age groups and this was statistically significant when compared age group of 45 to 60 year old (*p* = 0.04) (Fig 2A). No statistical difference was found among the other groups, despite the fact that the 45–60 year olds seem to have a higher level of HBV DNA VL.

According to CD4 cell count groups, HBV DNA VL was higher among patients with CD4 cell counts less than 200 cells/mm^3^ than those with CD4 cell counts greater than 500 cells/mm^3^ (*p* = 0.008) (Fig 2B).

No statistical difference was found in the level of HBV DNA VL between treated and untreated patients (Fig 2C). Furthermore, with regard to the anti HBV molecules, there was no statistically significant difference among patients received TDF-based therapy, patients received 3TC-based therapy and or untreated patients (Fig 2D).

However, the percentage of undetectable HBV DNA VL was statistically higher in treated group (72.3%) than in untreated (57.1%) (p = 0.0007) (Table 3). According to the therapeutic combinations, no statistical difference was found between monotherapy 3TC *versus* bitherapy TDF-3TC/FTC (*p* = 0.58).

**Table 3:** Rate of detectable and undetectable HBV DNA, among the treated and untreated groups, and comparing different therapeutic combination.

	Treated (n = 148)		Untreated (n = 35)	
	HBV DNA VL			
	Detectable	Undetectable	Detectable	Undetectable
**Any treatment combined**	27.7% 41/148	**72.3%** (107/148)	42.9% (15/35)	**57.1%** (20/35)
	X2 test between 72.3 vs 57.1: ***p* = 0.0007**			
**3TC Mono-therapy** (**n = 49**)	24.5% (12/49)	**75.5%** (37/49)	X2 test between: 75.5% vs 70.9% (*p* = 0.58)	
**Bi-therapy TDF-3TC/FTC** (**n = 79**)	29.1% (23/79)	**70.9%** (56/79)		

HBV: hepatitis B virus; 3TC: Lamivudine; TDF: Tenofovir; FTC: Emtricitabine; HBV DNA VL: viral load of nucleic acid of hepatitis B virus; n: number of patients.

### HBV genotype distribution

Among 56 patients with HBV DNA, 10 (18.5%) samples were amplified in the S gene and among them, 7 were amplified in the C gene. All the sequenced HBV strains were obtained from patients with an HBsAg^Pos^ profile and HBV DNA VL > 2000 copies/mL. The non-amplified samples (39; 84.8%) had HBV DNA VL less than 2000 copies/mL. Seven (15.2%) of samples with HBV DNA VL more than 2000 copies/mL failed to amplify.

**Fig 3:**
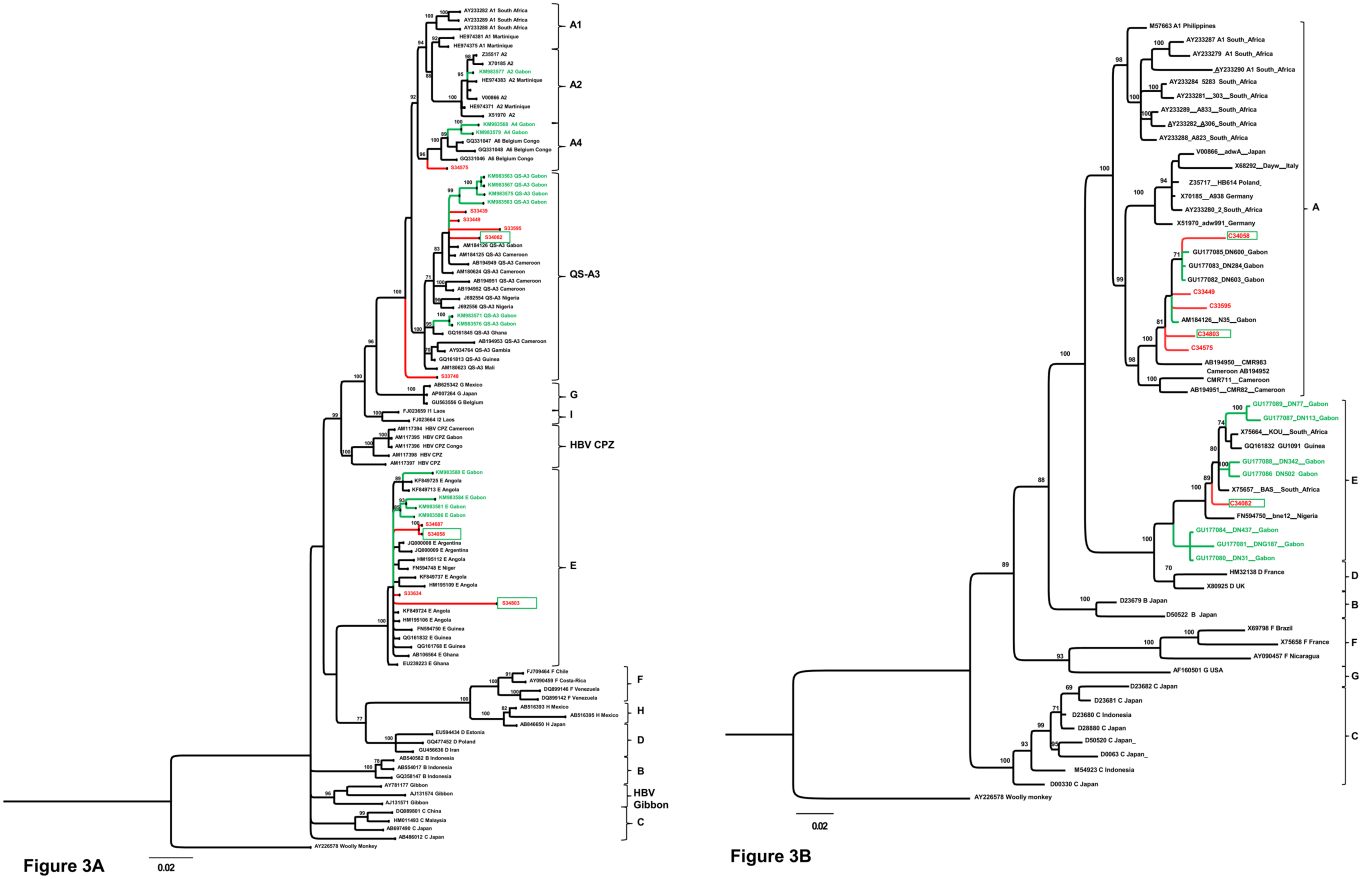
Phylogenetic analysis of partial surface gene (Fig 3A) and core gene (Fig 3B) rooted on HBV woolly monkey strain (AY226578). This tree was inferred by the Bayesian method in the GTR model. Samples sequenced in this study are represented in red. The green sequences correspond to Gabonese sequences recently described. The green framed sequences correspond to the potential recombinant (GenBank accession number of referenced strains, Genotypes and Countries of origin are indicated in the tree). Only Branching Posterior Probability (BPP) values ≥ 70% are illustrated.

Sequence analysis revealed that 6/10 (60%; 95% CI [29.6%-90.4%]) of strains belonged to the HBV-A genotype. Four HBV strains clustered with sub-genotype QS-A3 and 2 HBV strains with sub-genotype A4, all closely related to Cameroon and Gabon HBV strains. Four HBV strains (40%; 95% CI [6.96%-70.4%]) belonged to HBV-E genotype and were closely related to Gabon, Guinea and Angola HBV strains (Fig 3A).

Analysis of gene C shows that 5/6 (83%) strains clustered with HBV-A genotype and 1/6 strains with HBV-E genotype (Fig 3B). The tree strains (N°34058, 34082 and 34803), showing different classification in the two genes studied, could be considered as recombinant. All HBV sequences described herein were submitted to GenBank (accession numbers from KY271377 to KY271392).

### Resistance associated with mutations (RAMs) and Virus escape mutations (VEMs)

**Fig 4:**
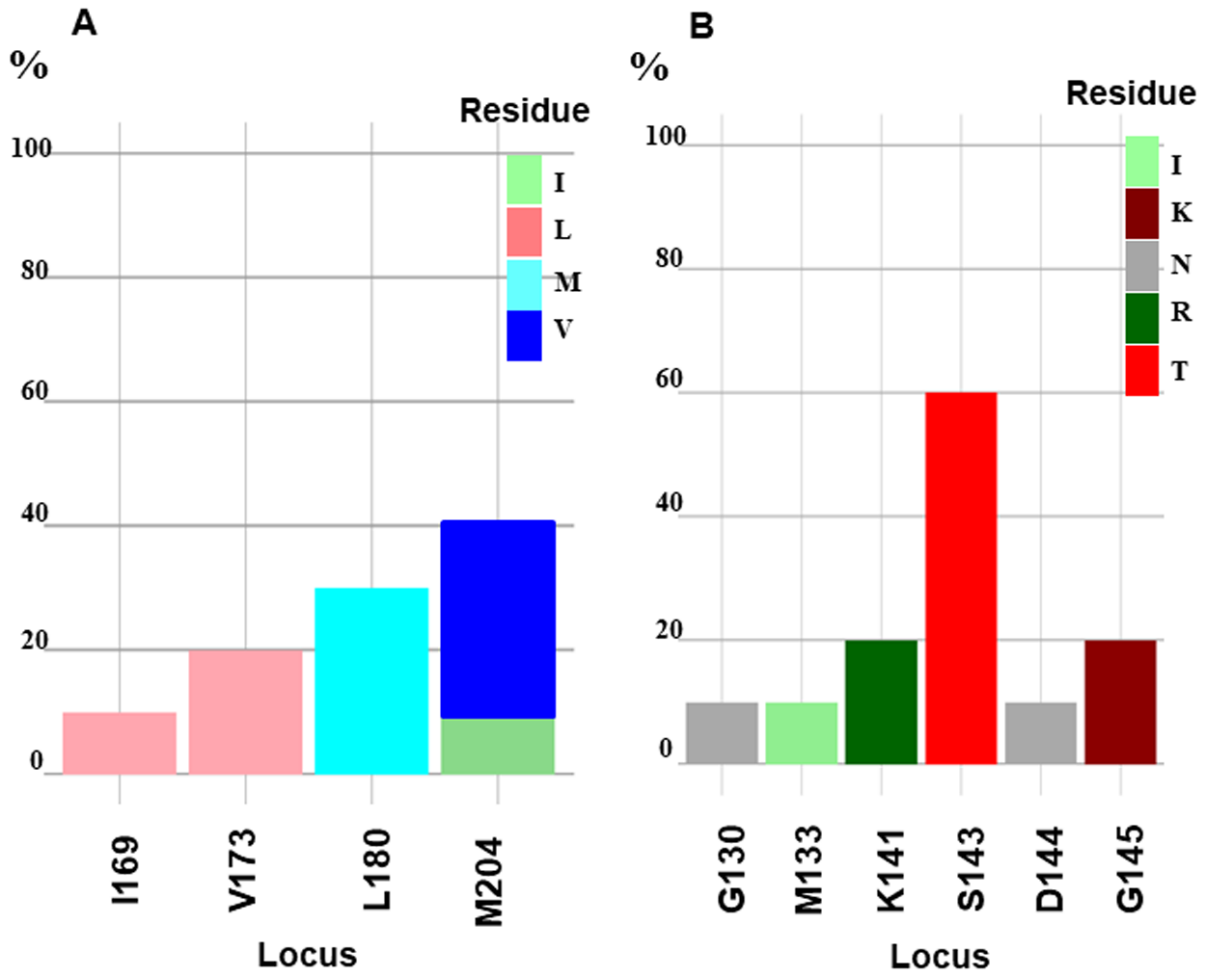
Distribution of mutations in the Polymerase gene (A) and in S gene (B) using Mutation Reporter Tool (http://hvdr.bioinf.wits.ac.za/tools/). Letters preceding each locus on the X axis show the reference patterns and the letters indexed in colors show the mutated patterns. Only detected mutations are represented.

The analysis of HBV strains for the S/Pol gene by the MRT online software showed that of the major RAMs, M204V/I was the most frequent (4/10, 40%), followed by its compensatory RAMs; L180M (3/10, 30%) and V173L (2/10, 20%). The MRT revealed I169L mutation in one patient (Fig 4, Table 4).

**Table 4:** Patients characteristics corresponding to the sequenced strains.

ID	Sex/ Age	Treatment	HBV DNA Viral Load (copies/mL)	CD4 (cells/mm^3^)	Mutations		Genotypes	
					Pol gene	S gene	S gene	C gene
33439	F/37	Untreated	20,300,000	NA	Wild Type	S143T	QS-A3	Field
34575	F/30	Untreated	> 50,000,000	68	Wild Type	S143T	A4	A
33449	M/49	3TC-FTC-EFV	> 50,000,000	1	Wild Type	S143T	QS-A3	A
33595	M/53	3TC-AZT-EFV	> 50,000,000	66	**M204I**	**G130N** S143T	QS-A3	A
34082	M/44	TDF-FTC-EFV	41,000,000	7	Wild Type	S143T	QS-A3	E
33748	F/46	ABC-DDI-LPV/r TDF-3TC-EFV *	2,680	15	**L180M M204V**	S143T	A4	NA
33634	M/50	TDF-FTC-EFV	197,000	11	Wild Type	Wild Type	E	NA
33678	F/52	TDF-3TC-LPV/r	9,810	349	V173L **L180M M204V**	K141R G145K	E	NA
34803	F/32	TDF-FTC-EFV	> 50,000,000	90	I169L	M133I D144V	E	A
34058	F/49	3TC-AZT-NVP	> 50,000,000	294	V173L **L180M M204V**	K141R G145K	E	A

ID: Identity; M: male; F: female; ABC: Abacavir; AZT: Zidovudine; DDI: Didanosine; EFV: Efavirenz; FTC: Emtricitabine; LPV/r: Lopinavir/Ritonavir; TDF: Tenofovir; 3TC: Lamivudine.

*: Old treatment. In bold, major mutation with clinical implication in resistance to ARVs and immune system escape. In red, discordant genotype between S and C gene highlighting a possible recombinant strain.

All patients with RAM M204V/I received 3TC-based therapy. There was no associations between HBV genotype and RAM mutations (Table 4). Looking for VEMs, MRT showed only one patient highlighted the VEM G130N. However, 60% of our strains had the S143T polymorphic mutation followed by G145K and K141R (for 2 patients) and M133I and D144N (for one patient) (Fig 4, Table 4).

## Discussion

The purpose of this study was to generate new data on the prevalence and genetic diversity of HBV infection among HIV-infected individuals in Libreville.

In the studied population, 8.8% of PLHA were positive for HBsAg in the same order of magnitude as previously describe in Franceville (9.3%) and in Cameroon (9.8%) [17,28]. This result is comparable to those previously reported in HIV-infected patients in Africa ranging from 8.3% to 23.7% (30).The prevalence was higher in men, in patients more than 30 years old, and increased with age. This difference could probably be due to specific risk factors such as alcohol abuse or sexual behavior with multiple partners [29]. Moreover, more than half of these HBsAgPos patients had CD4 cell counts <200/mm3. This corroborates the fact that most immunocompromised patients are unable to control their HBV infection [30,31], and/or these patients may experience HBV reactivation [32]. Our data confirm those found in Franceville and thus these characteristics appear to be a common feature of the Gabonese population. Indeed, the detection rate of HBV DNA in HBsAgPos patients in this study (69.7%; 30/43) was comparable to that reported previously in Franceville (76.1%; 54/71) [17] and also reported in Cameroon where 57,4% (31/54) of HBsAg positive patients had an HBV DNA level >40 IU/ml [28].

In this study, all non-amplified patients had HBV DNA VL <2000 copies/mL. For the others we obtained 100% amplification. Real-time PCRs are known to be much more sensitive than nested PCRs. In order to improve sensitivity, it will be necessary to increase the amount of plasma extracted or use specific universal primer designed to detect HBV DNA [33]. This is a limitation in our study, and in the upcoming studies, we should have to consider this in order to determine the genotypes of these strains. On the other hand, levels of HBV DNA VL, in persons chronically infected (HBsAg^Pos^), showed no significant difference between treated and untreated patients, as we found in Franceville [17]. With regard to rate of undetectable HBV DNA VL in treated compare to untreated, this study confirms the attempt of undetectable level of HBV therapy [34]. However, no difference was found between 3TC monotherapy based and bitherapy TDF-3TC/FTC. This leads us to discuss about TDF failure. Multiple studies showed the real effectiveness of TDF at one year until five years of treatment [34–36]. But some studies highlighted the mains risk factors of TDF failure like high HBV DNA VL, low CD4 cell count, anterior treatment with 3TC and the management of treatment [31,37]. In the context of this study, the majority of patients had a high HBV VL at anti-HBV started treatment and low CD4 cells count. Moreover, the difficulty in TDF supply was a real thing before and during the study period in Gabon. In fact, in case of breakage, switching to monotherapy 3TC is common. This management of ARV, mainly encountered in RLS, may have a real impact in TDF failure and has a negative impact in HBV DNA VL suppression [38]. Moreover, the high rate of HBV DNA VL and the previous treatment by 3TC regimen at baseline of the TDF start regimen in our patients seems to be factor of this failure as documented in the literature [37,38].

The prevalence of OBI found in this study was higher than that reported in PLHA in Cameroon (5.9%) [39], but lower than that found in Botswana (26.5%) [40]. This difference is probably related to different sensitivities of the amplification techniques used in these two studies (nested PCR in Cameroon, less sensitive *versus* real-time PCR in Botswana, more sensitive) HBV DNA was found in one patient without any serological marker of HBV infection. This patient was 42 years old (with probably past HBV resolved infection), has 16 CD4 cells/mL and combined with their HIV status, we can hypothesize about loss of antibodies against HBV virus, as supported by multiple studies [41–43]. This theory, if confirmed, would have serious consequences on HBV diagnosis. Finally, we did not find HBV DNA in patients harboring serological signature of vaccination (HBsAb^Pos^), which is strengthen the protective aspect of vaccination. By elsewhere, we should not rule out the rare cases of patients who have lost their HBsAg and HBcAb or even those falsely positive for HBsAb [44,45]. The sensitivity and specificity of ELISA test should be reassessed in Gabon [46].

The molecular analysis showed that a majority of HBV circulating strains in Libreville belonged to HBV-A genotype followed by HBV-E genotype. Within the HBV-A genotype, the HBV QS-A3 sub-genotype was the most encountered followed by HBV-A4 sub-genotype. These strains were previously described among PLHA and HIV uninfected individuals [17]. The ancient origin of HBV-A genotype in West and Central Africa has been demonstrated and it continues to spread. The HBV-E genotype, of more recent origin according to the authors [11], continues to expand in this part of the world. We have already demonstrated the presence of recombination between these two genotypes A and E [21]. In our study, we characterized 3 strains that may suggest recombination (1 QS-A3/E and 2 E/A). However, the hypothesis of multiple infections should not be ruled out as previously demonstrated [47].

We showed that, drug-resistant HBV also exist in Libreville, and they relate to patients being treated or previously treated with 3TC. For 3 patients, mutations M204V and M180V were present, for 1 patient only the M204I mutation was detected. All showed detectable HBV DNA VL. No relationship between genotype and presence of mutations was found, contrary to what was observed in Franceville [17]. A single HBV strain has an I169L mutation, at a position associated with resistance to Entecavir, although leucine (L) is not the amino acid involved [48]. However, to our knowledge, Entecavir is not currently available in Gabon. Finally, among our ten HBV sequenced strains, we found only one strain with a G130N mutation like VEM as described, and associated with diagnostic failure, rarely described (1%) [49]. However, we found 20% of patient with G145K and K141R mutations, usually incriminated in the immune escape [50], mainly in patients with mutations for 3TC resistance. The mutation S143T in our results was common to all HBV-A genotype strains. These particular genomic profiles need to be deepened.

Finally, our study was limited by the absence of clinical and biochemical parameters. Furthermore, in this study we have not ARV treatment duration at the inclusion. Further studies, are needed to evaluate the real impact of HBV treatment in Care Centers in the 9 provinces of Gabon.

## Conclusion

This study provides a new landmark in the global understanding of HIV-HBV co-infection in Gabon, with supplemental information on an urban area which is an important mix of different populations and culture (Libreville, capital of Gabon). It confirmed the high prevalence of HBsAg as well as occult HBV infections with the co-circulation of HBV genotypes A and E and possible recombinants strains. This study also indicates a high proportion of mutations conferring resistance to 3TC among HBV treated patients and a low rate of virus escape mutations. Further studies using a larger study population have to be conducted to confirm these results.

The presence of mutation and the low therapeutic efficacy are indicators of insufficient management of HBV infection. The causes must be investigated on a larger scale in the national territory. Finally, it is essential to improve the management of these patients co-infected with HIV and HBV, with a focus on widespread use of TDF in therapeutic combinations.

In view of our results, the diagnosis of OBI must be improved in the territory and their management should be investigated by the competent authorities.

## Supporting information

S1 Method Optimal extraction and particular amplification of HBV DNA from seronegative OBI sample. (XLSX)
